# Pro-inflammatory gene expression and neurotoxic effects of activated microglia are attenuated by absence of CCAAT/enhancer binding protein β

**DOI:** 10.1186/1742-2094-8-156

**Published:** 2011-11-10

**Authors:** Marco Straccia, Núria Gresa-Arribas, Guido Dentesano, Aroa Ejarque-Ortiz, Josep M Tusell, Joan Serratosa, Carme Solà, Josep Saura

**Affiliations:** 1 Biochemistry and Molecular Biology Unit, School of Medicine, University of Barcelona, IDIBAPS, Barcelona, Spain; 2 Department of Brain Ischemia and Neurodegeneration, IIBB-CSIC, IDIBAPS, Barcelona, Spain

## Abstract

**Background:**

Microglia and astrocytes respond to homeostatic disturbances with profound changes of gene expression. This response, known as glial activation or neuroinflammation, can be detrimental to the surrounding tissue. The transcription factor CCAAT/enhancer binding protein β (C/EBPβ) is an important regulator of gene expression in inflammation but little is known about its involvement in glial activation. To explore the functional role of C/EBPβ in glial activation we have analyzed pro-inflammatory gene expression and neurotoxicity in murine wild type and C/EBPβ-null glial cultures.

**Methods:**

Due to fertility and mortality problems associated with the C/EBPβ-null genotype we developed a protocol to prepare mixed glial cultures from cerebral cortex of a single mouse embryo with high yield. Wild-type and C/EBPβ-null glial cultures were compared in terms of total cell density by Hoechst-33258 staining; microglial content by CD11b immunocytochemistry; astroglial content by GFAP western blot; gene expression by quantitative real-time PCR, western blot, immunocytochemistry and Griess reaction; and microglial neurotoxicity by estimating MAP2 content in neuronal/microglial cocultures. C/EBPβ DNA binding activity was evaluated by electrophoretic mobility shift assay and quantitative chromatin immunoprecipitation.

**Results:**

C/EBPβ mRNA and protein levels, as well as DNA binding, were increased in glial cultures by treatment with lipopolysaccharide (LPS) or LPS + interferon γ (IFNγ). Quantitative chromatin immunoprecipitation showed binding of C/EBPβ to pro-inflammatory gene promoters in glial activation in a stimulus- and gene-dependent manner. In agreement with these results, LPS and LPS+IFNγ induced different transcriptional patterns between pro-inflammatory cytokines and NO synthase-2 genes. Furthermore, the expressions of IL-1β and NO synthase-2, and consequent NO production, were reduced in the absence of C/EBPβ. In addition, neurotoxicity elicited by LPS+IFNγ-treated microglia co-cultured with neurons was completely abolished by the absence of C/EBPβ in microglia.

**Conclusions:**

These findings show involvement of C/EBPβ in the regulation of pro-inflammatory gene expression in glial activation, and demonstrate for the first time a key role for C/EBPβ in the induction of neurotoxic effects by activated microglia.

## Background

Glial activation is an inflammatory process that occurs in astrocytes and microglia to re-establish homeostasis of the CNS after a disequilibrium of normal physiology. Microglia are tissue-associated macrophages that keep the CNS under dynamic surveillance. Most insults to the CNS switch microglia into an M1-like phenotype, characterized by production of pro-inflammatory cytokines, reactive oxygen/nitrogen species and prostanoids. Scavenger receptors and chemokines are also upregulated and phagocytic activity increases. An M2-like phenotype usually follows, characterized by production of interleukin-4 (IL-4), IL-10, transforming growth factor-β and neurotrophic factor [1]. Glial activation requires massive and fine-tuned re-arrangements in gene transcription. The transcription factors behind this process include nuclear factor-kB, which seems to mediate early-immediate cytokine and chemokine gene responses in glial activation [2,3], and other transcription factors with a pro-inflammatory profile such as AP-1 [4], STATs [5], HIF-1 [5-7], Egr-1 [8], IRF1 [9]. On the other hand, transcription factors such as PPARs [10] or Nrf2 [11,12] play an anti-inflammatory role in glial activation.

CCAAT/enhancer binding protein β (C/EBPβ) is a candidate to regulate pro-inflammatory gene expression in glial activation. C/EBPβ is one of seven members of the C/EBP subfamily of bZIP transcription factors. At least three N-terminally truncated isoforms are known: 38-kDa Full, 35-kDa LAP and 21-kDa LIP [13,14]. C/EBPβ transcriptional functions in cell energy metabolism, cell proliferation and differentiation are well-characterized [15,16]. C/EBPβ also plays a role in inflammation [17]. Promoters of many pro-inflammatory genes contain putative C/EBPβ consensus sequences [18-20] and C/EBPβ levels are upregulated in response to pro-inflammatory stimuli in macrophages [21] and glial cells [22-25]. Interestingly, C/EBPβ deficiency provides neuroprotection following ischemic [26] or excitotoxic injuries [27].

Several lines of evidence suggest that glial activation is involved in the pathogenesis of many neurological disorders. The present study stems from this hypothesis and from the hypothesis that there is a regulatory role for C/EBPβ in pro-inflammatory gene expression in neuroinflammation. To define the transcriptional role of C/EBPβ in glial activation we have here studied pro-inflammatory gene profiles and neurotoxicity in glial cultures from C/EBPβ-null mice. Our results show for the first time that absence of C/EBPβ attenuates pro-inflammatory gene expression and abrogates neuronal loss induced by activated microglia.

## Methods

### Animals

A colony of C/EBPβ^+/- ^[28] mice on a C57BL/6-129S6/SvEv background was maintained. Animals from this colony showed no serological evidence of pathological infection. The animals were group-housed (5-6) in solid floor cages and received a commercial pelleted diet and water ad libitum. Experiments were carried out in accordance with the Guidelines of the European Union Council (86/609/EU) and following the Spanish regulations (BOE 67/8509-12, 1988) for the use of laboratory animals, and were approved by the Ethics and Scientific Committees from the Hospital Clínic de Barcelona.

### DNA extraction and genotyping

Genomic DNA was isolated from 2 mg liver samples using Extract-N-AmpTissue PCR Kit (Sigma-Aldrich, XNAT2) following kit instructions. PCR amplification was performed in 20 μl total volume, using 1 μl of tissue extract, 0.8 μM C/EBPβ-1s forward primer (AAgACggTggACAAgCTgAg), 0.4 μM C/EBPβ-NeoAs (CATCAgAgCAgCCgATTgTC) and 0.4 μM C/EBPβ-4As (ggCAgCTgCTTgAACAAg TTC) reverse primers. Samples were run for 35 cycles (94°C for 30 s, 59°C for 30 s, 72°C for 90 s).

### Cortical mixed glial culture from a single embryo

C/EBPβ+/- mice were crossed and pregnant females were sacrificed on the 19th day of gestation by cervical dislocation. Embryos (E19) were surgically extracted from the peritoneal cavity. Their livers were dissected and used to genotype the animal, whereas their brains were dissected and processed as previously described [29] with minor modifications. Cultures reached confluence after 16 ± 3 days in vitro (DIV) and were then subcultured.

### Mouse mixed glial subculture

Each flask was washed in serum-free medium and was digested with 0.25% trypsin-EDTA solution for 5 min at 37°C. Trypsinization was stopped by adding an equal volume of culture medium with FBS 10%. Cells were pelleted (7 min, 180 g), resuspended in 1 mL culture medium, and brought to a single cell suspension by repeated pipetting. Cells were seeded at 166000 cells/mL. These were therefore secondary cultures and they were used at 12 ± 3 DIV. Astrocytes were the most abundant cell type and microglial cells were approximately 20%.

### Microglial culture

Microglial cultures were prepared by mild trypsinization from mouse mixed glial culture as previously described [30].

### Primary cortical neuronal culture

Cortical neuronal cultures were prepared from C57BL/6 mice at embryonic day 16 as described [31]. Neuronal cultures were used at 5 DIV.

### Primary neuronal-microglial co-cultures

Microglial cultures were obtained as described [31]. After astrocyte removal, microglial cells were incubated with 0.25% trypsin for 10 min at 37°C. Trypsinization was stopped by adding the same volume of culture medium with 10% FBS. Cells were gently scraped and centrifuged for 5 min at 200 g. Pellets were resuspended in neuronal culture medium and aliquots of the cell suspension (10 μL/well) were seeded on top of 5 DIV primary neuronal cultures at a final density of 4 × 10^5 ^cells/mL (1.3 × 10^5 ^cells/cm^2^).

### *In vitro *treatments

Mixed glial cultures: The culture medium was replaced 24 h prior to treatment. Mixed glial cultures were treated with 100 ng/mL lipopolysaccharide (LPS, Sigma-Aldrich, L-2654, E. coli serotype 026:B6) and 0.1 ng/mL recombinant mouse interferon-γ (IFNγ, Sigma-Aldrich, I4777) prepared from x10 solutions.

Neuronal-primary microglia co-cultures: 100 ng/mL LPS and 30 ng/mL IFNγ were added to the culture medium one day after seeding primary microglial cells on top of neuronal cultures.

### Nitrite assay

NO production was assessed by the Griess reaction. Briefly, 50 μL aliquots of culture supernatants were collected 48 h after LPS+IFNγ treatment, and incubated with equal volumes of Griess reagent (1% sulphanilamide, 0.1% N-(1-naphthyl)ethylendiamine dihydrochloride, and 5% phosphoric acid) for 10 min at room temperature (RT). Optical density at 540 nm was determined using a microplate reader (Multiskan spectrum, Thermo Electron Corporation). Nitrite concentration was determined from a sodium nitrite standard curve.

### Electrophoretic mobility shift assay

Nuclear extracts were prepared as described [32] with a few modifications. Nuclear protein was extracted from mixed glial cultures after 2 h LPS or LPS+IFNγ treatment. Cells from two wells of 6-well plate were scrapped into cold 0.01 M phosphate-buffered saline (PBS, pH 7.4) and centrifuged for 4 min, 4500 g at +4°C. The resulting pellet was resuspended in 400 μL of buffer A: 10 mM HEPES pH 7.9, 10 mM KCl, 0.1 mM EDTA, 0.1 mM EGTA, 0.5 mM phenylmethylsulphonyl fluoride (PMSF) and 1 mM dithiothreitol (DTT) and cells were swollen on ice for 15 min. After addition of 25 μL of 10% Igepal CA-630 (Sigma-Aldrich, I8896), cells were vigorously vortexed for 10 s and incubated for 10 min on ice, then a 10-min centrifugation at 13200 g was performed and the pellets were resuspended in 50 μL of buffer C consisting of 20 mM HEPES pH 7.9, 0.4 M NaCl, 1 mM EDTA, 1 mM EGTA, 1 mM PMSF and 1 mM DTT. Solutions A, B, C and PBS were supplemented with protease inhibitor cocktail Complete^® ^(Roche, 1836145). After 2 h of shaking at 4°C, nuclei were pelleted by a 5 min spin at 2000 g. The supernatant containing nuclear proteins was collected and protein amount was determined by the Lowry assay (Total Protein kit micro-Lowry, Sigma-Aldrich, TP0300). Oligonucleotides containing C/EBP consensus sequences (Santa Cruz Biotechnology, sc-2525) were labelled at their 3'-end using [α-^33^P]dATP (3000 Ci/mmol; Dupont-NEN, NEG-612H) and terminal deoxynucleotidyltransferase (TdT; Oncogene Research Products, PF060), and purified using illustra MicroSpin G-50 Columns (GE, 27-5330-01). Five micrograms of nuclear proteins were incubated for 30 min at RT with the labelled oligonucleotides (25000 cpm/reaction assay) in binding buffer (20% glycerol, 5 mM MgCl_2_, 2.5 mM EDTA, 2.5 mM DTT, 50 mM Tris-HCl, 250 mM NaCl and 0.2 mg/mL Poly(dI:dC)). After the addition of Hi-Density TBE buffer to samples (15% Ficoll type 400, 1x TBE, 0.1% Bromophenol Blue, 0.1% Xylene Cyanol), proteins were separated by electrophoresis on a 6% DNA retardation gel (Invitrogen, EC6365BOX) at 4°C, 90 min at 100 V in 0.5x TBE buffer. In supershift assay, 0.5 μg of rabbit anti-mouse C/EBPβ (Santa Cruz Biotechnology, sc-150) or IgG (Santa Cruz Biotechnology, No.sc-2027) were added 10 min before oligonucleotide incubation.

### Total protein extraction

Protein levels were determined in primary mixed glial cells 16 h after treatments. For isolation of total proteins, two wells from 6-well plates were used per condition. After a cold PBS wash, cells were scrapped and recovered in 100 μL per well of RIPA buffer (1% Igepal CA-630, 5 mg/mL sodium deoxycholate, 1 mg/mL sodium dodecyl phosphate (SDS) and protease inhibitor cocktail Complete^® ^in PBS). The content of the wells was pooled, sonicated, centrifuged for 5 min at 10400 g and stored at -20°C. Protein amount was determined by the Lowry assay.

### Western blot

Fifty micrograms of denatured (2.5 mM DTT, 100°C for 5 min) total protein extracts were subjected to 10% SDS-PAGE and transferred to a PVDF membrane (Millipore, IPVH00010) for 90 min at 1 mA/cm^2^. After washing in Tris-buffered saline (TBS: 20 mM Tris, 0.15 M NaCl, pH 7.5) for 5 min, dipping in methanol for 10 s and air drying, the membranes were incubated with primary antibodies overnight at 4°C: polyclonal rabbit anti-C/EBPβ (1:500, Santa Cruz Biotechnology, sc-150), monoclonal mouse anti-NO synthase-2 (NOS2; 1:200, BD Transduction Laboratories, 610431), monoclonal mouse anti-βactin (1:100000, Sigma-Aldrich, A1978) and polyclonal rabbit anti-GFAP (1:10000, DakoCytomation, Z0334) diluted in immunoblot buffer (TBS containing 0.05% Tween-20 and 5% no-fat dry milk). Then, the membranes were washed twice in 0.05% Tween-20 in TBS for 15 s and incubated in horseradish peroxidase (HRP)-labelled secondary antibodies for 1 h at RT: donkey anti-rabbit (1:5000, GE, NA934) or goat anti-mouse (1:5000, Santa Cruz Biotechnology, sc-2055). After extensive washes in 0.05% Tween-20 in TBS, they were incubated in ECL-Plus (GE, RPN2132) for 5 min. Membranes were then exposed to the camera of a VersaDoc System (Bio-Rad), and pixel intensities of the immunoreactive bands were quantified using the percentage adjusted volume feature of Quantity One 5.4.1 software (Bio-Rad). Data are expressed as the ratio between the intensity of the protein of interest band and the loading control protein band (β-actin).

### Quantitative real time PCR (qPCR)

mRNA expression was determined in mouse mixed glial cells 6 h after treatments. For isolation of total RNA, 2 wells of 24-well plates were used per experimental condition. Total RNA was isolated using an Absolutely RNA Miniprep kit (Agilent Technologies-Stratagene 400.800) and 100 ng of RNA for each condition was reverse-transcribed with random primers using Sensiscript RT kit (Qiagen, 205213). cDNA was diluted 1/25 and 3 μL were used to perform qPCR. The primers (Roche) were used at a final concentration of 300 nM (Table 1). β-Actin and Rn18s mRNAs levels are not altered by treatments (data not shown). qPCR was carried out with IQ SYBR Green SuperMix (Bio-Rad, 170-8882) in 15 μL of final volume using iCycler MyIQ equipment (Bio-Rad). Primer efficiency was estimated from standard curves generated by dilution of a cDNA pool. Samples were run for 40 cycles (95°C for 30 s, 60°C for 1 min, 72°C for 30 s). Amplification specificity was confirmed by analysis of melting curves. Relative gene expression values were calculated with the comparative Ct or ΔΔCt method [33] using iQ5 2.0 software (Bio-Rad). Ct values were corrected by the amplification efficiency of the respective primer pair which was estimated from standard curves generated by dilution of a cDNA pool.

**Table 1:** Primers used in quantitative real time PCR.

Target Gene	Accession	Primer forward (5→3')	Primer reverse (5→3')
NOS2	NM_010927.3	ggCAgCCTgTgAgACCTTTg	gCATTggAAgTgAAgCgTTTC
IL1β	NM_008361.3	TggTgTgTgACgTTCCCATTA	CAgCACgAggCTTTTTTgTTg
IL6	NM_031168.1	CCAgTTTggTAgCATCCATC	CCgCAgAggAgACTTCACAg
TNFα	NM_013693.2	TgATCCgCgACgTggAA	ACCgCCTggAgTTCTggAA
TGFβ1	NM_011577.1	TgCgCTTgCAgAgATTAAAA	AgCCCTgTATTCCgTCTCCT
IL4	NM_021283, 2	CgAggTCACAggAgAAgggA	AAgCCCTACAgACgAgCTCACT
Actin	NM_007393.3	CAACgAgCggTTCCgATg	gCCACAggATTCCATACCCA
Rn18s	NR_003286.2	gTAACCCgTTgAACCCCATT	CCATCCAATCggTAgTAgCg

### Quantitative chromatin immunoprecipitation (qChIP)

qChIP was performed as previously described [34] with modifications. Briefly, primary mixed glial cultures were cross-linked in 1% formaldehyde for 10 min at RT, quenched with 125 mM glycine for 5 min a RT. Cells were washed in PBS with 1 mM PMSF and protease inhibitor mix, then the cells were resuspended with 150 mM NaCl, 50 mM Tris-HCL pH7.5, 5 mM EDTA, 0.5% vol/vol NP-40, 1% vol/vol Triton X-100, 1% wt/vol SDS, 1 mM PMSF, protease inhibitor mix (IP Buffer). Chromatin shearing was obtained from 2 × 10^5 ^cells using Labsonic M sonicator (7 × 30 s on and 30 s off; cycle 0.8; 100% amplitude). In parallel, an aliquot of chromatin sheared from each sample was separated as a loading control for the experiment (input). The protocol for chromatin immunoprecipitation (ChIP) was as follows: first, 10 μL of Dynabeads^® ^protein A (Invitrogen, 100.01D) were washed twice with 22 μL of cold IP Buffer (without SDS). Then the beads were resuspended in 11 μL of IP Buffer. Next, 90 μL of IP Buffer was added to a PCR tube with 10 μL of pre-washed protein A-beads. Two micrograms of polyclonal rabbit C/EBPβ antibody (Santa Cruz Biotechnology, sc-150X) or with 2 μg of rabbit IgG (Santa Cruz Biotechnology, sc-2027) as negative control were added and the mixture was incubated at 40 rpm on a rotating wheel for at least 2 h at 4°C. Then, the tube was placed on a magnetic rack for 1 min. The supernatant was discarded and 100 μL of sheared chromatin was added. Samples were incubated overnight at 40 rpm rotation at 4°C. Finally, the tube was placed on the magnetic rack for 1 min. The supernatant was discarded and the immunoprecipitation complex was washed three times with 100 μL of IP Buffer for 4 min on a rotating wheel and placed in the magnetic rack again for 1 min to discard the supernatant. The fourth wash was done with 10 mM Tris-HCl pH 8.0 and 10 mM EDTA buffer. Protein was degraded by a 2-h incubation at 68°C in 200 μL of IP Buffer complemented with 50 μg/mL of proteinase K. DNA was isolated with phenol-chloroform-isoamylalcohol 25:24:1 (Sigma-Aldrich, 25666 and P4556) extraction. Input and ChIP samples were analyzed with qPCR using SYBR green (Bio-Rad). Three microliters of input DNA (diluted 1/50) and ChIP were amplified in triplicate in 96-well optical plates using a MyIQ Bio-Rad Real Time Detection System. The C/EBPβ binding site in the IL-10 promoter was used as a positive control [35]. MatInspector was used to identify the proximal C/EBPβ consensus sequence in each analyzed promoter. The sequences for each amplified locus are indicated in the table 2. Samples were run for 45 cycles (95°C for 30 s, 62°C for 1 min, 72°C for 30 s), for further details see qPCR methods.

**Table 2:** C/EBPβ binding sites and primers used in quantitative ChIP assay.

Target Gene	C/EBPβ binding site sequence (5→3') Consensus: ATTGCGCAAT	Genomic localization respect to ATG	Primer forward (5→3')	Primer reverse (5→3')
NOS2	ggagTGaaGCAATga	-892/-907	TTATgAgATgTgCCCTCTgC	CCACCTAAggggAACAgTgA
IL1β	tgtgTgaaGaAAgaa	-16/-31	TCAggAACAgTTgCCATAgC	AgACCTATACAACggCTCCT
IL6	gTttCCAATcagccc	-173/-188	gTTgTgATTCTTTCgATgCT	ggAATTgACTATCgTTCTTg
TNFα	agggTTtgGaAAgtt	-336/-351	TCTCATTCAACCCTCggAAA	CACACACACCCTCCTgATTg
IL10	aggATTGaGaAATaa	-463/-448	TgACTTCCgAgTCAgCAAgA	AgAggCCCTCATCTgTggAT

### Immunocytochemistry

Cultured cells were fixed with 4% paraformaldehyde in PBS for 20 min at RT. For immunocytochemistry using fluorescence labelling, cells were permeated with chilled methanol for 7 min, then washed with PBS. Cells were incubated overnight at 4°C with 7% normal goat serum (Vector, S-1000) in PBS containing 1% Thimerosal (Sigma-Aldrich, T5125) and primary antibodies: polyclonal rabbit anti-C/EBPβ (1:500, Santa Cruz Biotechnology, sc-150), monoclonal mouse anti-NOS2 (1:200, BD Transduction Laboratories, 610431), polyclonal rabbit anti-GFAP (1:1000, DakoCytomation, Z0334) and monoclonal rat anti-CD11b (1:300, Serotec, MCA711G, clone 5C6). After rinsing in PBS, cells were incubated for 1 h at RT with secondary antibodies: goat anti-mouse Alexa 546 (1:1000, Molecular Probes, A-11018), goat anti-rabbit Alexa 546 (1:1000, Molecular Probes A-11010), Alexa 488 (1:1000, Molecular Probes, A-11070) or goat anti-rat Alexa 488 (1:500, Molecular Probes, A-11006). After secondary antibody incubation, cells were stained with Hoechst 33258 for 7 min. For immunocytochemistry using peroxidase labelling, cells were permeated and endogenous peroxidase activity was blocked by incubation with 0.3% H_2_O_2 _in methanol for 10 min. Non-specific staining was blocked by incubating the cells with 10% normal goat serum in PBS containing 1% BSA for 20 min at RT. The cells were then incubated with monoclonal mouse anti-MAP2 primary antibody (1:2000, Sigma-Aldrich, M1406) overnight at 4°C. In MAP2 staining, biotinylated horse anti-mouse secondary antibody (1:200, Vector, BA-2000) for 1 h at RT. Following incubation with ExtrAvidin^®^-Peroxidase (1:500, Sigma-Aldrich, E2886) for 1 h at RT, colour was developed with diaminobenzidine (Sigma-Aldrich, D5637). The antibodies were diluted in PBS containing 1% BSA and 10% normal horse serum (Vector, S-2000). Microscopy images were obtained with an Olympus IX70 microscope and a digital camera (CC-12, Soft Imaging System GmbH).

### Assessment of neuronal viability (MAP2/ABTS/ELISA)

Neuronal viability was evaluated by MAP2 immunostaining using ABTS (2, 3'-azinobisethylbenzothiazoline-6-sulphonic acid) and absorbance analysis [31]. Neuronal viability was expressed as a percentage of control levels.

### Cell counting

Hoechst-33258- and CD11b-positive cells were semi-automatically counted from 20x photomicrographs using ImageJ 1.42I NIH software. For each experiment (n = 4), three wells per condition were used and four fields per well were counted in a blind manner. NOS2-positive cells were counted manually from 20x photomicrographs. For each experiment (n = 11), two wells per condition were used and two fields per well were counted.

### Statistical analysis

Data were analyzed using GraphPad 4.02. Two-way analysis of variance (ANOVA) followed by Bonferroni post-test was used when the effect of genotype on treatment was studied and vice versa. One-way ANOVA was used followed by Dunnet's post-test when comparing versus control or Bonferroni's post-test when comparing versus different experimental conditions. Values of p < 0.05 were considered statistically significant. Error bars are presented in all graphs as standard error of the mean (SEM).

## Results

### Characterization of C/EBPβ^+/+ ^and C/EBPβ^-/- ^single embryo secondary mixed glial cultures

**Figure 1:**
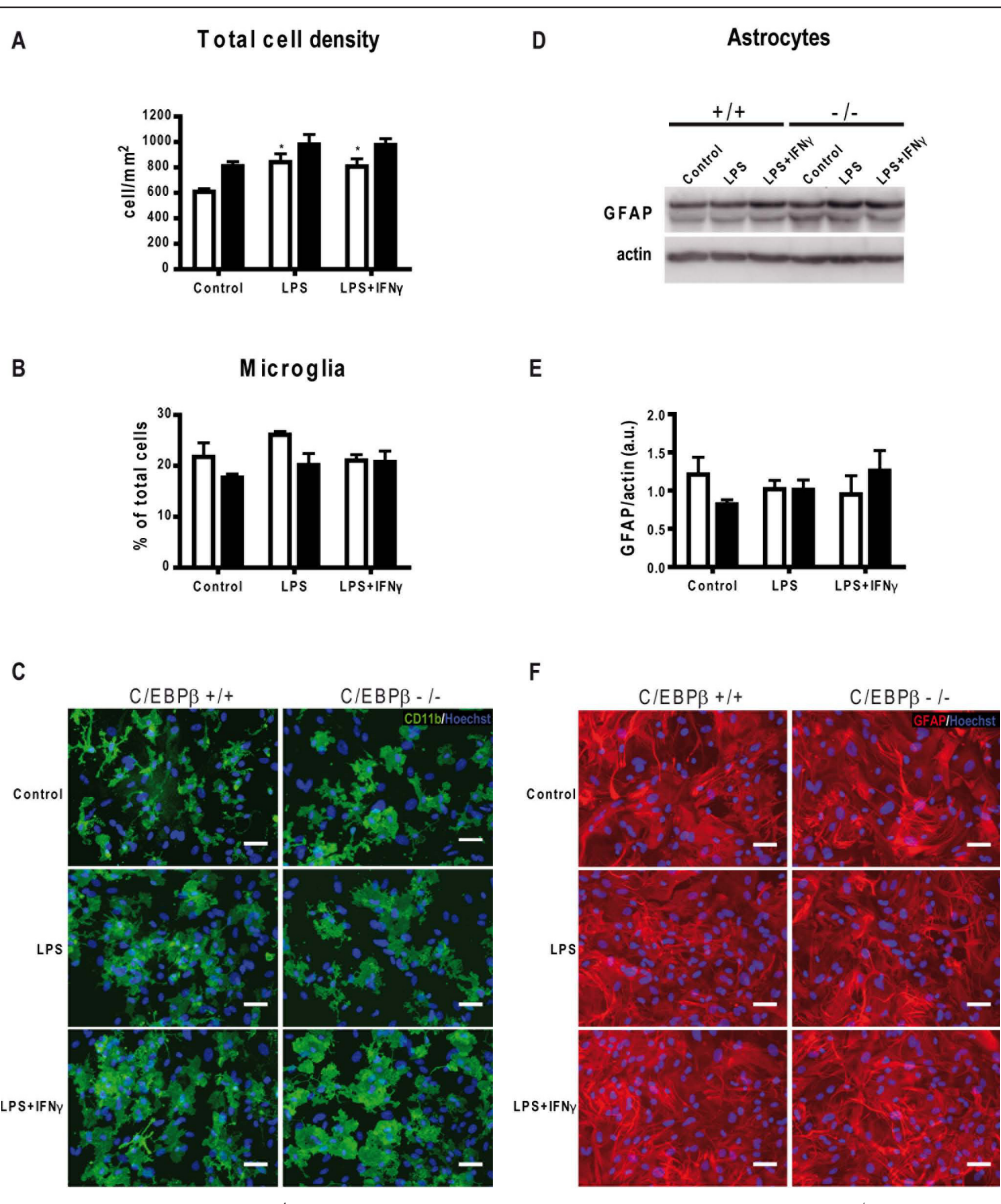
**Basic characterization of C/EBPβ^-/- ^mixed glial cultures**. Secondary mixed glial cultures from C/EBPβ^+/+ ^(white bars) and C/EBPβ^-/- ^(black bars) show similar total cell numbers and microglial density in control conditions and after 16 h of LPS or LPS+IFNγ. **A**. C/EBPβ^+/+ ^and C/EBPβ^-/- ^total cell density was estimated by Hoechst-33258-positive nucleus counting. No significant differences were observed between genotypes. Wild-type cultures show a statistically significant increase of cell density after 16 h of LPS and LPS+IFNγ treatment compared to control; C/EPBβ-null cultures show no difference after treatments. Two-way ANOVA, followed by Bonferroni's test was applied. *p < 0.05; compared to C/EBPβ^+/+ ^control. (n = 4). **B**. Microglia as a percentage of total cells was estimated by CD11b-positive cell counting in C/EBPβ^+/+ ^and C/EBPβ^-/- ^cultures after 16 h treatments with LPS, LPS+IFNγ or vehicle. Significant differences among treatments groups or genotypes are not observed. Two-way ANOVA, followed by Bonferroni's test was applied. (n = 4). **C**. Secondary mixed glial cultures were immunostained for CD11b (green). Nuclei are stained with Hoechst-33258 (blue). Microglial cell numbers were similar for C/EBPβ^+/+ ^and C/EBPβ^-/- ^cultures. LPS and LPS+IFNγ induced morphological changes in microglial cells in both genotypes. Bar = 50 μm. **D**. A representative western blot shows levels of GFAP in C/EBPβ^+/+ ^and C/EBPβ^-/- ^mixed glial protein extracts 16 h after vehicle (control), LPS and LPS+IFNγ treatments. β-Actin was used for normalization. **E**. Densitometric analysis was used to quantify GFAP protein levels versus β-actin in 4 independent western blots in arbitrary units (a.u.). Changes in GFAP protein levels are not observed. Two-way ANOVA, followed by Bonferroni's test was applied. (n = 4). **F**. Secondary mixed glial cultures were immunostained for GFAP (red) showing a confluent astrocytic layer. Overlapping of astroglial cell bodies makes counting very difficult and imprecise. No differences in astroglial morphology or density among genotypes are observed. Nuclei are stained with Hoechst-33258 (blue). Bar = 50 μm. **E**. Lack of NOS2 expression in activated astrocytes. C/EBPβ^+/+ ^and C/EBPβ^-/- ^secondary mixed glial cultures were immunostained for GFAP (green) and NOS2 (red), and stained for Hoechst 33258 (blue), after 16 h of LPS+IFNγ treatment. A marked reduction in number of NOS2-positive cells is seen in C/EBPβ-null cultures. The representative merge images show clearly that NOS2-positive cells do not colocalize with GFAP-positive cells. Bar = 50 μm.

To study the role of C/EBPβ in glial activation we used C/EBPβ-null mice. Because of the infertility of C/EBPβ-null females and a perinatal death rate of approximately 50% for C/EBPβ-null neonates, we have modified the standard procedures to prepare mixed glial cultures from CNS tissue pools of several mouse neonates and designed a protocol to prepare secondary mixed glial cultures from the cerebral cortex of one single E19-E20 mouse embryo (see Methods for details). Forty-one C/EBPβ-null mice and forty-one wild-type littermates were used during this study. To ensure that wild-type and C/EBPβ-null glial cultures were comparable, we first analyzed total cell density and abundance of their two main cell types, astrocytes and microglia, in both cultures. No differences were observed between wild-type and C/EBPβ-null cultures in total cell density as assessed by automatic counting of Hoechst 33258-stained nuclei (Figure 1A), but a moderate increase in total cell number was induced by LPS and LPS+IFNγ. C/EBPβ absence did not affect microglial density as assessed by CD11b-positive cell counting (Figure 1B). Estimation of astrocytes number in these cultures is not trivial. Astrocytes are densely packed, almost all nuclei are surrounded by GFAP-positive filaments, and it is often difficult to discern whether a given nucleus belongs to a GFAP-positive cell or, in fact, the GFAP signal belongs to a neighbor astrocyte. We therefore analyzed total GFAP content by western blot as an indirect estimation of astroglial number and no differences were observed between wild-type and C/EBPβ-null glial cultures (Figure 1D, E). Neither CD11b nor GFAP immunocytochemistry revealed differences between wild-type or C/EBPβ-null cultures in morphology of microglial cells or astrocytes, respectively (Figure 1C, F). These results indicate that wild-type and C/EBPβ-null mixed glial cultures do not differ in total cell density or in proportions or morphology of their two major cell types, astrocytes and microglia.

### LPS and LPS+IFNγ upregulate C/EBPβ in secondary mixed glial cultures

**Figure 2:**
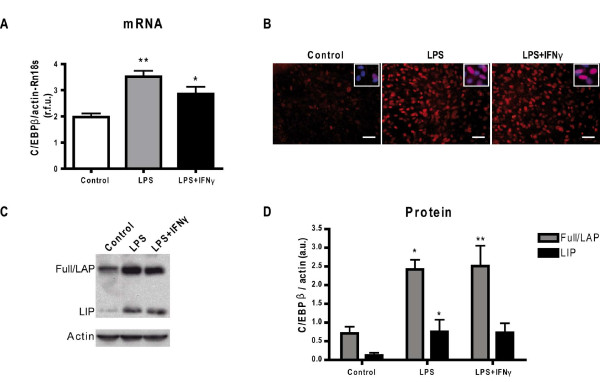
**C/EBPβ expression in activated mixed glial cultures**. Effect of 100 ng/mL LPS alone or in combination with 0.1 ng/mL IFNγ on C/EBPβ expression in secondary mixed glial cultures. **A**. C/EBPβ mRNA expression is upregulated in glial activation. Cultures were treated with LPS and LPS+IFNγ for 6 h and mRNA was analyzed by qPCR. Results are expressed as relative fold units (r.f.u.) of ΔΔCt values between C/EBPβ and actin + Rn18s as reference genes. One-way ANOVA followed by Dunnett's test is applied. *p < 0.05; **p < 0.01 compared to control. (n = 3). **B**. LPS and LPS + IFNγ (24 h) increase nuclear C/EBPβ immunostaining (red) in secondary mixed glial cultures. In the right upper corner, a detail shows overlapping between Hoechst 33258 nuclear staining and C/EBPβ. Images are representative of 5 independent experiments. Bar = 50 μm. **C**. A western blot shows levels of C/EBPβ in secondary mixed glial cultures treated with LPS or LPS + IFNγ for 24 h. The C/EBPβ isoforms are identified as Full/LAP and LIP. β-Actin is used for normalization. This experiment was done 4 times with similar results. **D**. Full/LAP (grey bars) upregulation after LPS and LPS+IFNγ is statistically significant compared to control. LIP (dashed bars) upregulation is statistically significant only for LPS treatment. One-way ANOVA, followed by Dunnett's test is applied. *p < 0.05; **p < 0.01 compared to respective control. (n = 4-5)

In this study, we have used LPS and LPS+IFNγ to study the role of C/EBPβ in glial activation in secondary cultures. The effects of both stimuli on C/EBPβ expression in glial cultures have not been compared before. As seen in Figure 2A-D, both LPS and LPS+IFNγ induced strong increases in C/EBPβ mRNA levels 6 h after treatment, and in nuclear levels of both activating (Full/LAP) and inhibitory (LIP) C/EBPβ isoforms 24 h after treatment. The increases in C/EBPβ mRNA and protein induced by LPS and LPS+IFNγ were of similar magnitude.

### Differential C/EBPβ activation is triggered by LPS and LPS+IFNγ

**Figure 3:**
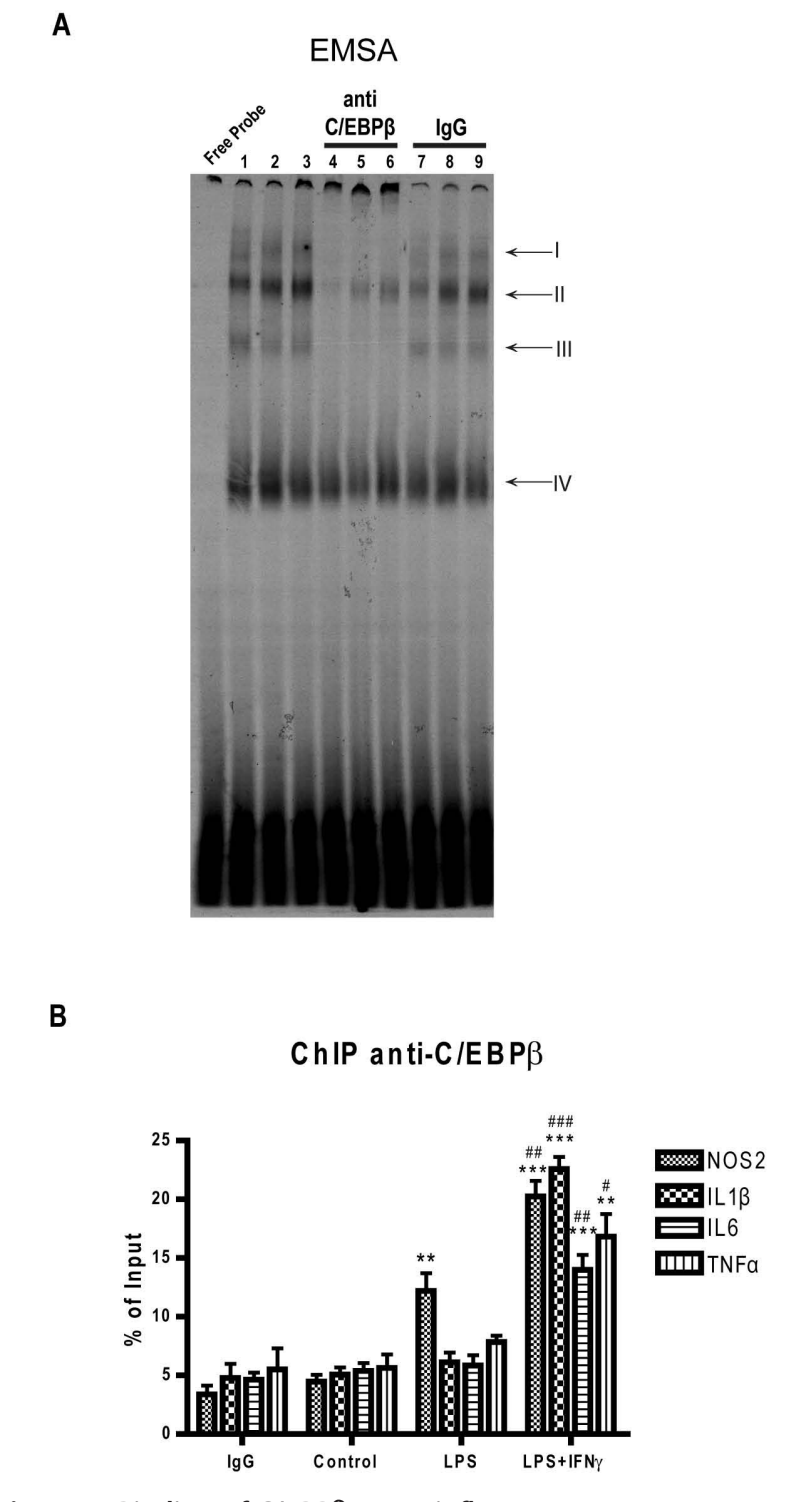
**Binding of C/EBPβ to proinflammatory gene promoters in activated mixed glial cultures**. **A**. C/EBPβ DNA binding activity was analyzed by gel shift and supershift assays. Nuclear proteins were extracted from secondary mixed glial cultures treated with vehicle (lanes 1, 4, 7), LPS (lanes 2, 5, 8) or LPS+IFNγ (lanes 3, 6, 9) for 2 h. The first lane represents the probe without nuclear extract incubation (free probe). Arrows indicate four shifted complexes. Complex IV is a C/EBPβ independent complex. Lanes 1 to 3 show C/EBPs shifting complexes in wild type condition. Supershift with anti-C/EBPβ antibody (lanes 4 to 6) shows the presence of C/EBPβ in I-III complexes in all treatments. Rabbit IgG (lanes 7 to 9) is used as negative control for the supershift assay. This image is representative of four independent experiments. **B**. Quantitative analysis of C/EBPβ binding to NOS2, IL-1β, IL-6 and TNFα promoters by qChIP in mixed glial cultures. The sequences and positions of every C/EBPβ binding site and the primers used for qPCR are found in table 2. IL-10 was used as positive control. The qChIP assay was carried out after 2 h of LPS, LPS+IFNγ or vehicle (control) treatment. The IgG bars represent the means for IgG/Control, IgG/LPS and IgG/LPS+IFNγ PCR values for each gene. Input refers to total DNA. % of input represents the percentage of qChIP/Input ratio. One-way ANOVA, followed by Bonferroni's multiple comparison test is applied. **p < 0.01; ***p < 0.001 compared to control. #p < 0.05; ##p < 0.01; ###p < 0.001 compared to LPS. (n = 3)

Since the mRNA or protein levels of a transcription factor are of relative importance to study its functionality, we studied the DNA binding activity of C/EBPβ in LPS- or LPS+IFNγ-treated glial cells. Electrophoretic mobility shift assays showed that binding of nuclear proteins to a DNA oligonucleotide containing the C/EBPs consensus sequence was increased by LPS and LPS+IFNγ treatments (Figure 3A, lanes 1-3). Supershift experiments showed the presence of C/EBPβ in shifted complexes I to III (Figure 3A lanes 4-6). The specificity of the supershift is demonstrated by the lack of supershift elicited by the same concentration of IgG (Figure 3A lanes 7-9). This indicates that C/EBPβ is a key component of C/EBPs DNA binding complexes during LPS- and LPS+IFNγ-induced glial activation.

Next, we estimated the binding of C/EBPβ to the promoters of four major pro-inflammatory genes: nitric oxide synthase 2 (NOS2), IL-1β, IL-6 and TNFα, in mixed glial cultures using a qChIP assay (Figure 3B). In untreated glial cultures, no specific binding of C/EBPβ was measurable in any of the four promoters analyzed. However, 2 h after LPS treatment, C/EBPβ binding was observed in the NOS2 promoter. Interestingly, in LPS+IFNγ-treated glial cultures C/EBPβ binding was observed in all four promoters analyzed and, in the case of the NOS2 promoter, C/EBPβ binding was significantly higher than in LPS-treated glial cultures (Figure 3B).

### C/EBPβ regulates pro-inflammatory gene expression in glial activation

**Figure 4:**
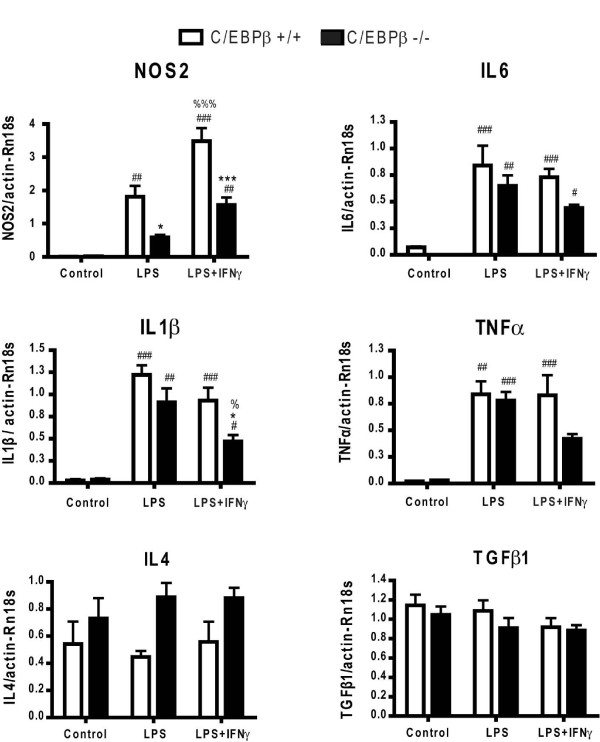
**Reduced proinflammatory gene expression in C/EBPβ^-/- ^mixed glial cultures**. Expression of pro-inflammatory (NOS2, IL-1β, IL-6 and TNFα) and anti-inflammatory (IL-4 and TGFβ1) genes in C/EBPβ^+/+ ^(white bars) and C/EBPβ^-/- ^(black bars) mixed glial cultures. Cultures were treated with LPS or LPS+IFNγ for 6 h and then mRNA levels were analyzed by qPCR. In wild type cultures LPS and LPS+IFNγ induce expression of the four pro-inflammatory genes studied but do not affect mRNA levels of the anti-inflammatory genes IL-4 and TGFβ1. Absence of C/EBPβ results in significant decreases in LPS-induced expression of NOS2 and in LPS+IFNγ-induced expression of NOS2 and IL-1β. Results are expressed as relative fold units of ΔΔCt value between gene of interest and actin + Rn18s as reference genes. Two-way ANOVA, followed by Bonferroni's test was applied. *p < 0.05, ***p < 0.001 compared to respective C/EBPβ^+/+ ^condition. ^#^p < 0.05; ^##^p < 0.01; ^###^p < 0.001 compared to respective control. ^%^p < 0.05; ^%%%^p < 0.001 compared to respective LPS condition.

To study the involvement of C/EBPβ in the regulation of pro-inflammatory gene expression, mRNA levels of NOS2, IL-1β, IL-6 and TNFα were analyzed by qPCR in wild-type and C/EBPβ-null cultures treated with LPS or LPS+IFNγ for 6 h. In wild-type cultures all four mRNAs were strongly upregulated by LPS. This effect was exacerbated by co-treatment with IFNγ in the case of NOS2 (+92.3%), but not in the case of IL-1β, IL-6 or TNFα (Figure 4). In C/EBPβ-null cultures LPS induced upregulation of IL-1β, IL-6 and TNFα mRNAs, which was similar to that observed in wild-type cultures. However, as expected from qChIP results, the LPS-induced increase in NOS2 mRNA levels was significantly lower in C/EBPβ-null than in wild-type glial cultures (-67.4%, p < 0.05). The pattern of gene expression induced by LPS+IFNγ was more affected by lack of C/EBPβ. Thus, LPS+IFNγ-induced mRNA levels of NOS2 and IL-1β were significantly lower in C/EBPβ-null than in wild-type cultures. TNFα and IL-6 mRNA levels did not differ statistically between the two genotypes (Figure 4). In contrast to the pro-inflammatory gene pattern, mRNA levels of the anti-inflammatory cytokines IL-4 and transforming growth factor β (TGFβ1) were not altered by LPS or LPS+IFNγ treatments and no significant changes in IL-4 or TGFβ1 mRNA levels were observed between wild-type and C/EBPβ-null glial cultures under any experimental condition (Figure 4).

### C/EBPβ-null glial cultures show a marked reduction in NO production

**Figure 5:**
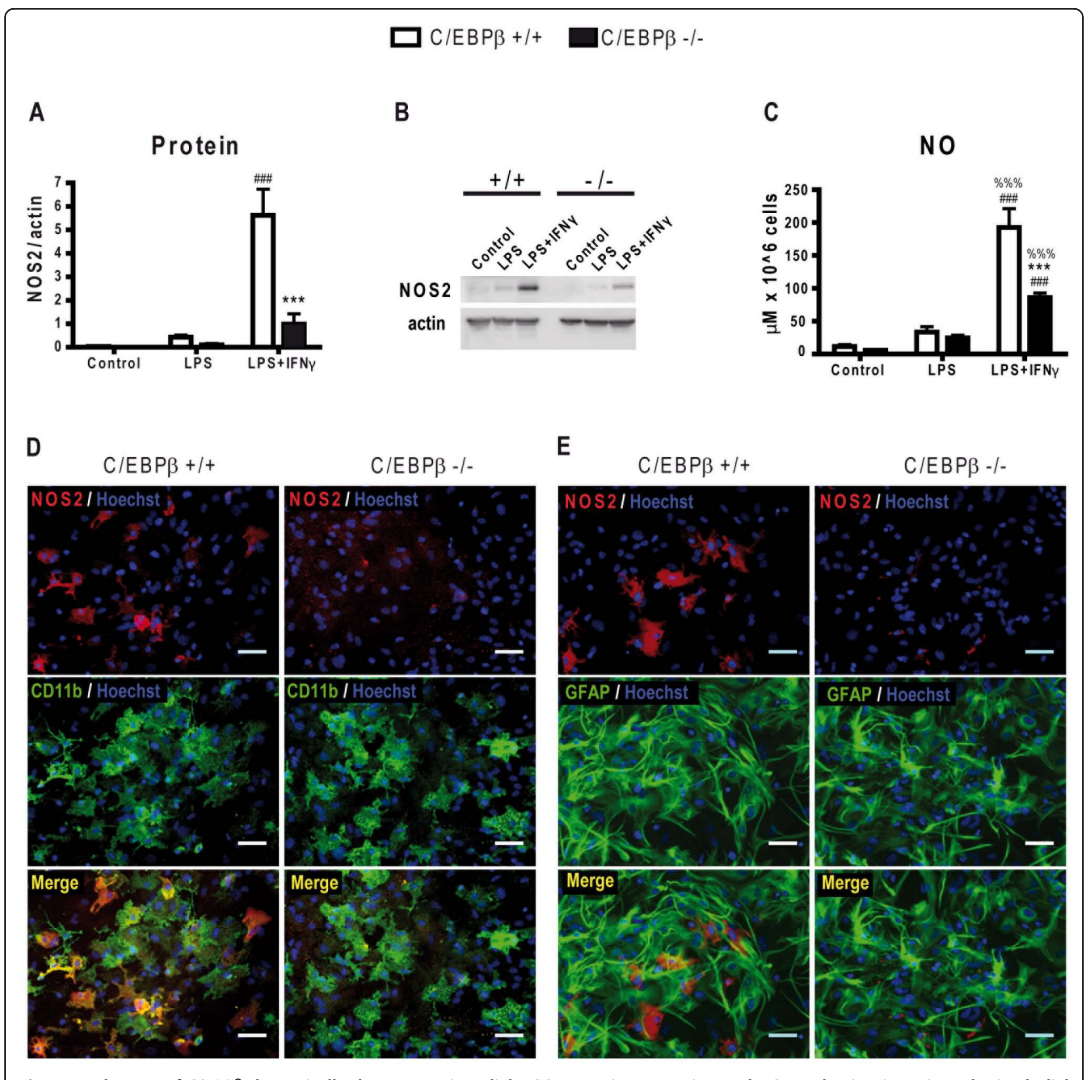
**Absence of C/EBPβ dramatically decreases microglial NOS2 protein expression and NO production in activated mixed glial cultures**. **A**. NOS2 protein levels in total protein extracts from mixed glial cultures were analyzed by western blot, followed by densitometry. Data is expressed as NOS2 versus β-actin band intensities. Cultures were treated for 16 h with LPS, LPS+IFNγ or vehicle. In C/EBPβ^+/+ ^cultures (white bars) NOS2 protein levels were detected after LPS treatment, but LPS+IFNγ induced a clear upregulation, in agreement with mRNA expression levels. In C/EBPβ^-/- ^mixed glial cultures (black bars), NOS2 protein levels decreased in LPS and LPS+IFNγ compared to C/EBPβ^+/+ ^cultures. Two-way ANOVA, followed by Bonferroni's test was applied. ***p < 0.001 compared with C/EBPβ^+/+ ^condition. ^###^p < 0.001 compared with respective control condition. (n = 5). A representative western blot is shown in **B**. **C**. NO production is decreased in activated C/EBPβ^-/- ^glial cultures. NO levels were measured by colorimetric analysis 48 h after treatments and normalized per cell number. Values are reported as micromolar concentration ×10^6 ^cells. NO levels in C/EBPβ^+/+ ^(white bars) cultures were upregulated after LPS and LPS+IFNγ treatments compared to controls. In C/EBPβ^-/- ^glial cultures (black bars), NO production is reduced in LPS+IFNγ treatment compared to wild-type NO levels. Two-way ANOVA, followed by Bonferroni's test was applied. ***p < 0.001 compared to C/EBPβ^+/+ ^condition. ^###^p < 0.001 compared to respective control condition. ^%%%^p < 0.001 compared to respective LPS condition. (n = 7-9). **D**. NOS2 is expressed by activated microglia. C/EBPβ^+/+ ^and C/EBPβ^-/- ^secondary mixed glial cultures were immunostained for CD11b (green) and NOS2 (red), and stained for Hoechst 33258 (blue) after 16 h of LPS+IFNγ treatment. The C/EBPβ^+/+ ^merged image shows colocalization of NOS2-positive cells and CD11b-positive cells. Bar = 50 μm.

The important reduction in NOS2 mRNA levels in activated C/EBPβ-null glial cultures prompted us to analyze NOS2 protein levels by western blot and immunocytochemistry, and generation of NO by colorimetric detection of nitrites (Griess assay). In wild-type cultures NOS2 protein expression was induced by LPS and more markedly by LPS+IFNγ. In C/EBPβ-null cultures LPS-induced NOS2 levels were not significantly different from wild-type whereas LPS+IFNγ-induced NOS2 protein levels were markedly reduced (-77.4%, p < 0.0001) (Figure 5A, B). NO levels correlated well with the NOS2 protein data and a strongly significant attenuation in NO production induced by LPS+IFNγ was seen in C/EBPβ-null cultures (Figure 5C).

The reduction in LPS+IFNγ-induced NOS2 expression in C/EBPβ-null glial cultures seen by western blot was confirmed by immunocytochemistry. We did not observe by immunocytochemistry any NOS2-positive cells in untreated cultures (not shown), whereas in LPS- (not shown) and LPS+IFNγ-treated wild-type cultures, NOS2 immunoreactivity was observed in 14.0 ± 3.6% of total cells (Figure 5D, E). The vast majority of NOS2-positive cells in LPS+IFNγ-treated wild type mixed glial cultures also expressed CD11b (99.3 ± 1.4%; n = 11) and very rarely NOS2-positive cells expressed GFAP (0.6 ± 1.2%; n = 11) indicating that in these conditions NOS2 expression in mouse cortical mixed glial cultures is predominantly microglial. In C/EBPβ-null cultures the number of NOS2 cells was dramatically reduced after either LPS (not shown) or LPS+IFNγ treatments (Figure 5D, E). As seen in Figure 5D, the reduction of NOS2-positive cells could not be attributed to a reduction in microglial density.

### C/EBPβ deficiency in activated microglia abrogates neurotoxicity

**Figure 6:**
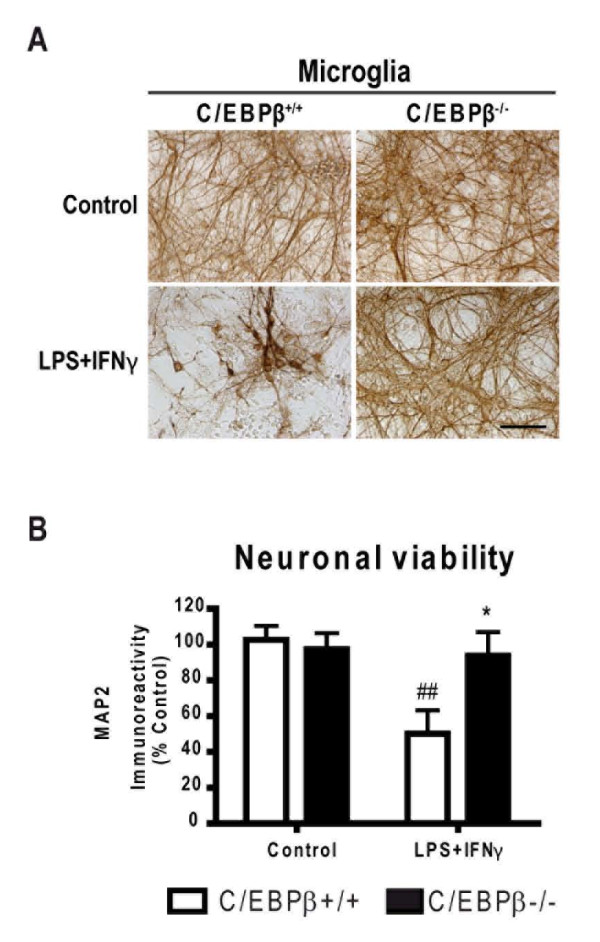
**Lack of C/EBPβ in activated microglia completely abolishes the neurotoxic effects of activated microglia in neuronal-microglial co-cultures**. **A**. MAP2 immunostaining of wild-type neurons co-cultured with C/EBPβ^+/+ ^or C/EBPβ^-/- ^microglia was performed 48 h after LPS+IFNγ treatment. MAP2 staining shows a clear decrease of network fibres caused by activated C/EBPβ^+/+ ^microglia, but not by C/EBPβ^-/- ^microglia or by vehicle-treated microglia. Images are representative of 5 independent experiments. (Bar = 100 μm). **B**. Evaluation of neuronal viability by MAP2/ABTS/ELISA assay 48 h after treatment with LPS+IFNγ or vehicle (control). Results are presented as % of MAP2 immunostaining in control cultures. Treatment with LPS+IFNγ reduces MAP2 immunostaining in neurons cocultured with C/EBPβ^+/+ ^microglia, but not with C/EBPβ^-/- ^microglia. Two-way ANOVA, followed by Bonferroni's test was applied. ^##^p < 0.01 compared with C/EBPβ^+/+ ^control; *p < 0.05 compared to C/EBPβ^+/+ ^LPS+IFNγ. (n = 5).

Activated microglia have strong neurotoxic potential [36]. The observations of reduced expression of pro-inflammatory mediators in LPS+IFNγ-activated C/EBPβ-null glial cells, particularly microglia, prompted us to analyze whether the neurotoxic effects of LPS+IFNγ-activated microglia could be attenuated by C/EBPβ absence. To this aim, wild-type and C/EBPβ-null microglial cells were isolated and co-cultured with wild-type neurons. No neuronal death was observed when neurons not co-cultured with microglia were treated with LPS+IFNγ or when neuron/wild-type microglia co-cultures were treated with LPS alone (data not shown). In contrast, LPS+IFNγ treatment of neuron/wild-type microglia co-cultures resulted in death of 51.2% of neurons, as estimated by MAP2/ABTS/ELISA (Figure 6). Interestingly, in neuron/C/EBPβ-null microglia co-cultures treated with LPS+IFNγ, MAP2 immunoreactivity levels were equal to control levels (Figure 6) indicating that the neurotoxicity induced by LPS+IFNγ-treated microglia was completely abolished in the absence of C/EBPβ. In this model, NO production plays a major role in the neurotoxicity elicited by activated microglia since the NOS2 inhibitor 1400W (10 μM) completely abolished neuronal death in LPS+IFNγ-treated neuron/microglia co-cultures (Gresa-Arribas et al, unpublished observations).

## Discussion

The transcription factor C/EBPβ is expressed in glia but no direct evidence exists for its involvement in glial activation. In the present study we show that both LPS and LPS+IFNγ upregulate C/EBPβ expression in mixed glial cultures to a similar extent. Both stimuli also induce C/EBPβ binding to proinflammatory gene promoters but this binding is stronger when induced by LPS+IFNγ. Lack of C/EBPβ results in attenuated expression of proinflammatory genes and, again, this effect is more pronounced when glial cells are activated with LPS+IFNγ than when LPS alone is the activating stimulus. Finally, we describe for the first time that neurotoxicity elicited by LPS+IFNγ-treated microglial cells is completely abrogated by lack of C/EBPβ.

In this study we have used mixed glial cultures composed mainly of astrocytes and microglia. This culture system is our model of choice to study glial activation because it allows cross-talk between the two cell types, which is extremely important in glial activation [37]. Working with astrocytes or microglia in isolation may yield misleading results and there are numerous examples of astroglial or microglial responses that are markedly affected by the absence of the other cell type [37-39]. Regarding C/EBPβ, we have previously shown in experiments with mixed glial and astroglial- or microglial-enriched cultures that, upon activation, C/EBPβ is primarily expressed by microglia with a lesser upregulation in astrocytes [24]. This suggests that the data here reported on C/EBPβ in glial activation mainly reflects C/EBPβ changes in microglia although part of the observed effects could be of astroglial origin. However, in the case of the effects of C/EBPβ absence on NOS2 expression and neurotoxicity, the observed effects are clearly microglial, as shown by the microglial localization of NOS2 immunoreactivity and by the use of isolated microglia, respectively.

Most protocols to prepare primary mixed glial cultures from rodents use pools of tissue from several neonates, generally one or two litters. Since C/EBPβ females are sterile [40] litters of C/EBPβ-null neonates cannot be obtained. Furthermore, approximately 50% of C/EBPβ-null pups die perinatally [28] which favors the use of late embryos instead of neonates to ensure a maximum number of available C/EBPβ-null mice. Therefore, we established for this study a new protocol of secondary mixed glial cultures by subculturing primary glial cultures prepared from the cerebral cortex of a single E19-E20 embryo. The use of secondary cultures was particularly suitable for this project because we could prepare mixed glial cultures that were very similar to primary cultures in terms of cell density and proportions with a more-than-2-fold higher yield. Besides, the use of siblings eliminates any genetic background effect. Altogether, this makes the use of secondary mixed glial cultures from a single embryo or neonate a useful approach when working with mouse strains of compromised fertility.

LPS is a toll-like receptor 4 agonist that induces marked changes in gene expression in astrocytes and microglia [1]. The combination of LPS, a pathogen factor, with IFNγ, a host factor, potentiates some of the LPS-induced effects [41]. Here we report for the first time a proper comparison between LPS and LPS+IFNγ effects on C/EBPβ and on pro-inflammatory markers in glial cells. We have observed that both LPS and LPS+IFNγ induce similar increases in C/EBPβ mRNA and protein levels as well as in DNA binding. Time-course analyses have revealed that upregulation of the C/EBPβ activating isoforms Full/LAP often precedes upregulation of the inhibitory isoform LIP [21,24,42]. When a single time-point is analyzed, as in the present study, the simultaneous increase in activating and inhibitory C/EBPβ isoforms is a common observation. EMSA analysis with supershift experiments showed the presence of C/EBPβ in bands I, II and III. These bands may contain different C/EBPβ isoforms (Full, LAP or LIP) with various post-translational modifications (phosphorylation, SUMOylation or acetylation has been described [43]). It is likely that some of these bands contain more than one complex (e.g. band II since it is only partially supershifted by anti-C/EBPβ) and that some of these complexes contain other transcription factors, p65-NFκB [44] and C/EBPδ [45,46] being two of the most likely candidates to form complexes with C/EBPβ in neuroinflammation. An extensive biochemical analysis would be necessary to characterize the transcriptional C/EBPβ complexes in activated glial cells.

This study shows for the first time in glial cells an analysis of mRNA levels for the pro-inflammatory genes NOS2, IL-1β, IL-6 and TNFα, comparing LPS and LPS+IFNγ as activating stimuli. In this model, IFNγ alone did not trigger any effect (data not shown) whereas LPS and LPS+IFNγ upregulated all four pro-inflammatory genes analyzed. LPS and LPS+IFNγ increased expression of IL-1β, IL-6 and TNFα to the same extent, as reported for macrophages [47], whereas LPS-induced upregulation of NOS2 was markedly potentiated by cotreatment with IFNγ, in agreement with previous observations in microglia [48] and macrophages [19]. Even though transcriptional levels of cytokine genes in LPS-treated glial cultures are not modulated by cotreatment with IFNγ, their promoter regions undergo a remodeling of transcriptional complex as proved by qChIP assay. mRNA analysis showed that absence of C/EBPβ does not affect LPS-induced upregulation of the three cytokines, in agreement with absence of C/EBPβ binding to IL-1β, IL-6 or TNFα promoters in LPS-treated glial cultures, as seen by qChIP. Although we cannot exclude the presence of C/EBPβ in other promoter regions, because we focused our promoter analysis on the C/EBPβ consensus sequence most proximal to the translation start site, these data strongly suggest that C/EBPβ does not participate in the LPS-induced expression of these three genes in the present model. It may seem contradictory that strong C/EBPβ binding to IL-1β, IL-6 and TNFα promoters was induced by LPS+IFNγ, but not by LPS alone, whereas the levels of these cytokine mRNAs were similar after treatment with either LPS or LPS+IFNγ. In our opinion, this indicates that different sets of transcription factors act on these promoters after LPS or LPS+IFNγ treatment or, in other words, that there is IFNγ-induced chromatin remodeling on these promoters [49]. This is also suggested by the qPCR data showing that LPS+IFNγ-induced expression of IL-1β is reduced in the absence of C/EBPβ, and that there is also a tendency toward reduced expression of TNFα and IL-6. These data demonstrate for the first time that C/EBPβ plays a role in transactivation of pro-inflammatory cytokine genes in glial cells induced by LPS+IFNγ but not by LPS alone.

In our glial activation model, the NOS2 gene shows a different transcription pattern when compared with the pro-inflammatory cytokines. On the one hand, as mentioned before, LPS-induced NOS2 expression is potentiated by co-treatment with IFNγ. On the other hand, C/EBPβ binding to the NOS2 promoter is already seen after LPS treatment alone and, interestingly, this binding is potentiated by IFNγ treatment. As observed in macrophage cell lines, IFNγ can trigger C/EBPβ phosphorylation, modulating its capacity to form transcriptional complexes with p300 [50] or Med1 [51]. Also, IFNγ can promote C/EBPβ DNA binding activity to IFN-stimulated regulatory elements (ISREs) which we have found tightly associated with C/EBPβ consensus sequences on the mouse NOS2 promoter (unpublished observations). Finally, both LPS- and LPS+IFNγ-induced increases in NOS2 expression are attenuated in the absence of C/EBPβ. These findings suggest that C/EBPβ plays a functional role both in LPS-induced NOS2 expression and in the potentiation of this effect elicited by IFNγ. In accordance with the multiple stage glial activation model [52], we can hypothesize that LPS alone activates the glia, but that only with a host warning signal, such as IFNγ, are glia totally committed to a hyper-reactive phenotype. We propose that C/EBPβ could trigger this shift through the executive phase of glial activation.

The hypothesis of a pathogenic role for exacerbated glial activation, particularly activation of microglia, is based on the known in vitro neurotoxic effects of activated microglia [53,54], on the protective effects of anti-inflammatory treatments or genetic modifications in animal models of neurodegenerative disorders [55,56] and on epidemiological data [57-59]. Since we have shown in this study that C/EBPβ deficiency attenuates expression of potentially neurotoxic pro-inflammatory mediators but not that of anti-inflammatory cytokines, we were interested to test the hypothesis that C/EBPβ plays a key role in the induction of detrimental effects by microglial activation. Reduced neuronal damage after ischemic [26] or excitotoxic insults [27] has been observed in C/EBPβ-null mice. Even though C/EBPβ expression has been reported in activated glial cells [22-24], C/EBPβ is known to be also expressed in the adult mouse by neurons [60] and peripheral cells [16]. Consequently, the neuroprotective effect observed in C/EBPβ-null mice could be mediated by lack of C/EBPβ in any of these cells. We show here that the neurotoxicity elicited by activated wild-type microglial cells co-cultured with wild-type neurons is completely abolished by the absence of C/EBPβ specifically in microglia. This strongly supports a role of C/EBPβ in the regulation of potentially neurotoxic effects of microglia and suggests that the neuroprotective effects of total C/EBPβ absence in vivo [26,27] are due to microglial C/EBPβ deficiency. Specific microglial C/EBPβ deletion would be very informative to clarify the role of microglial C/EBPβ in neurodegeneration in *in vivo *models of neurological disease.

## Conclusions

In summary, this study shows that LPS and LPS+IFNγ induce expression of C/EBPβ in mixed glial cultures, and both stimuli also induce differential binding of C/EBPβ to proinflammatory gene promoters. A functional role for C/EBPβ in glial activation is demonstrated by the attenuated gene expression and abrogation of neurotoxicity in microglial cells devoid of C/EBPβ. Altogether, these findings point to C/EBPβ as a key transcription factor in the molecular reprogramming that occurs in microglial activation and suggest that C/EBPβ is a possible therapeutic target to ameliorate neuronal damage of neuroinflammatory origin.

## List of abbreviations

ABTS: 2, 3'-azinobisethylbenzothiazoline-6-sulphonic acid; ANOVA: Analysis of variance; C/EBPβ: CCAAT/enhancer binding protein β; DIV: Days in vitro; GFAP: Glial fibrillary acidic protein; HRP: Horseradish peroxidase; IFNγ: Interferon γ; IL: Interleukin; LPS: Lipopolysaccharide; NOS2: NO synthase-2; qChiP: Quantitative chromatin immunoprecipitation; qPCR: Quantitative real time PCR; RT: Room temperature; TGFβ1: Transforming growth factor β1; TNFα: Tumour necrosis factor-α

## Competing interests

The authors declare that they have no competing interests.

## Authors' contributions

MS carried out most experiments and drafted the manuscript. NGA carried the experiments involving neuron/microglia cocultures. GD carried out the qChIP experiments. AEO set the C/EBPβ-null colony and carried out the preliminary experiments. JMT participated in the preparation of primary cultures. JSe participated in immunocytochemistry experiments. CS designed and participated in the neuron/microglia cocultures experiments and participated in the statistical analysis. JSa conceived and coordinated the study and drafted the manuscript. All authors critically revised and approved the final manuscript.

## References

[B1] Michelucci A Heurtaux T Grandbarbe L Morga E Heuschling P Characterization of the microglial phenotype under specific pro-inflammatory and anti-inflammatory conditions: Effects of oligomeric and fibrillar amyloid-beta J Neuroimmunol 2009 210 3 12 10.1016/j.jneuroim.2009.02.003 19269040

[B2] Cho IH Hong J Suh EC Kim JH Lee H Lee JE Lee S Kim CH Kim DW Jo EK Lee KE Karin M Lee SJ Role of microglial IKKbeta in kainic acid-induced hippocampal neuronal cell death Brain 2008 131 3019 3033 10.1093/brain/awn230 18819987 PMC2577806

[B3] Kaltschmidt B Widera D Kaltschmidt C Signaling via NF-kappaB in the nervous system Biochim Biophys Acta 2005 1745 287 299 10.1016/j.bbamcr.2005.05.009 15993497

[B4] Kwon D Fuller AC Palma JP Choi IH Kim BS Induction of chemokines in human astrocytes by picornavirus infection requires activation of both AP-1 and NF-kappa B Glia 2004 45 287 296 10.1002/glia.10331 14730702 PMC7165560

[B5] Kim OS Park EJ Joe EH Jou I JAK-STAT signaling mediates gangliosides-induced inflammatory responses in brain microglial cells J Biol Chem 2002 277 40594 40601 10.1074/jbc.M203885200 12191995

[B6] Wang X Li C Chen Y Hao Y Zhou W Chen C Yu Z Hypoxia enhances CXCR4 expression favoring microglia migration via HIF-1alpha activation Biochem Biophys Res Commun 2008 371 283 288 10.1016/j.bbrc.2008.04.055 18435916

[B7] Zhang W Petrovic JM Callaghan D Jones A Cui H Howlett C Stanimirovic D Evidence that hypoxia-inducible factor-1 (HIF-1) mediates transcriptional activation of interleukin-1beta (IL-1beta) in astrocyte cultures J Neuroimmunol 2006 174 63 73 10.1016/j.jneuroim.2006.01.014 16504308

[B8] Friedle SA Brautigam VM Nikodemova M Wright ML Watters JJ The P2X7-Egr pathway regulates nucleotide-dependent inflammatory gene expression in microglia Glia 2011 59 1 13 10.1002/glia.21071 20878769 PMC2981661

[B9] Iadecola C Salkowski CA Zhang F Aber T Nagayama M Vogel SN Ross ME The transcription factor interferon regulatory factor 1 is expressed after cerebral ischemia and contributes to ischemic brain injury J Exp Med 1999 189 719 727 10.1084/jem.189.4.719 9989987 PMC2192924

[B10] Drew PD Xu J Storer PD Chavis JA Racke MK Peroxisome proliferator-activated receptor agonist regulation of glial activation: relevance to CNS inflammatory disorders Neurochem Int 2006 49 183 189 10.1016/j.neuint.2006.04.003 16753239

[B11] Lee JM Li J Johnson DA Stein TD Kraft AD Calkins MJ Jakel RJ Johnson JA Nrf2, a multi-organ protector? FASEB J 2005 19 1061 1066 10.1096/fj.04-2591hyp 15985529

[B12] Shih AY Johnson DA Wong G Kraft AD Jiang L Erb H Johnson JA Murphy TH Coordinate regulation of glutathione biosynthesis and release by Nrf2-expressing glia potently protects neurons from oxidative stress J Neurosci 2003 23 3394 3406 12716947 10.1523/JNEUROSCI.23-08-03394.2003 PMC6742304

[B13] Ossipow V Descombes P Schibler U CCAAT/enhancer-binding protein mRNA is translated into multiple proteins with different transcription activation potentials Proc Natl Acad Sci USA 1993 90 8219 8223 10.1073/pnas.90.17.8219 8367486 PMC47320

[B14] Welm AL Timchenko NA Darlington GJ C/EBPalpha regulates generation of C/EBPbeta isoforms through activation of specific proteolytic cleavage Mol Cell Biol 1999 19 1695 1704 10022857 10.1128/mcb.19.3.1695 PMC83963

[B15] Greenbaum LE Li W Cressman DE Peng Y Ciliberto G Poli V Taub R CCAAT enhancer- binding protein beta is required for normal hepatocyte proliferation in mice after partial hepatectomy J Clin Invest 1998 102 996 1007 10.1172/JCI3135 9727068 PMC508965

[B16] Ramji DP Foka P CCAAT/enhancer-binding proteins: structure, function and regulation Biochem J 2002 365 561 575 12006103 10.1042/BJ20020508 PMC1222736

[B17] Poli V The role of C/EBP isoforms in the control of inflammatory and native immunity functions J Biol Chem 1998 273 29279 29282 10.1074/jbc.273.45.29279 9792624

[B18] Caivano M Gorgoni B Cohen P Poli V The induction of cyclooxygenase-2 mRNA in macrophages is biphasic and requires both CCAAT enhancer-binding protein beta (C/EBP beta) and C/EBP delta transcription factors J Biol Chem 2001 276 48693 48701 10.1074/jbc.M108282200 11668179

[B19] Lowenstein CJ Alley EW Raval P Snowman AM Snyder SH Russell SW Murphy WJ Macrophage nitric oxide synthase gene: two upstream regions mediate induction by interferon gamma and lipopolysaccharide Proc Natl Acad Sci USA 1993 90 9730 9734 10.1073/pnas.90.20.9730 7692452 PMC47644

[B20] Reddy ST Wadleigh DJ Herschman HR Transcriptional regulation of the cyclooxygenase-2 gene in activated mast cells J Biol Chem 2000 275 3107 3113 10.1074/jbc.275.5.3107 10652293

[B21] Bradley MN Zhou L Smale ST C/EBPbeta regulation in lipopolysaccharide-stimulated macrophages Mol Cell Biol 2003 23 4841 4858 10.1128/MCB.23.14.4841-4858.2003 12832471 PMC162211

[B22] Cardinaux JR Allaman I Magistretti PJ Pro-inflammatory cytokines induce the transcription factors C/EBPbeta and C/EBPdelta in astrocytes Glia 2000 29 91 97 10.1002/(SICI)1098-1136(20000101)29:1<91::AID-GLIA9>3.0.CO;2-I 10594926

[B23] Cardinaux JR Magistretti PJ Vasoactive intestinal peptide, pituitary adenylate cyclase-activating peptide, and noradrenaline induce the transcription factors CCAAT/enhancer binding protein (C/EBP)-beta and C/EBP delta in mouse cortical astrocytes: involvement in cAMP-regulated glycogen metabolism J Neurosci 1996 16 919 929 8558260 10.1523/JNEUROSCI.16-03-00919.1996 PMC6578805

[B24] Ejarque-Ortiz A Medina MG Tusell JM Perez-Gonzalez AP Serratosa J Saura J Upregulation of CCAAT/enhancer binding protein beta in activated astrocytes and microglia Glia 2007 55 178 188 10.1002/glia.20446 17078024

[B25] Perez-Capote K Saura J Serratosa J Sola C Expression of C/EBPalpha and C/EBPbeta in glial cells in vitro after inducing glial activation by different stimuli Neurosci Lett 2006 410 25 30 10.1016/j.neulet.2006.09.078 17070994

[B26] Kapadia R Tureyen K Bowen KK Kalluri H Johnson PF Vemuganti R Decreased brain damage and curtailed inflammation in transcription factor CCAAT/enhancer binding protein beta knockout mice following transient focal cerebral ischemia J Neurochem 2006 98 1718 1731 10.1111/j.1471-4159.2006.04056.x 16899075

[B27] Cortes-Canteli M Luna-Medina R Sanz-Sancristobal M Alvarez-Barrientos A Santos A Perez-Castillo A CCAAT/enhancer binding protein beta deficiency provides cerebral protection following excitotoxic injury J Cell Sci 2008 121 1224 1234 10.1242/jcs.025031 18388310

[B28] Screpanti I Romani L Musiani P Modesti A Fattori E Lazzaro D Sellitto C Scarpa S Bellavia D Lattanzio G Bistoni F Frati L Cortese R Gulino A Ciliberto G Constantini F Poli V Lymphoproliferative disorder and imbalanced T-helper response in C/EBP beta-deficient mice EMBO J 1995 14 1932 1941 7744000 10.1002/j.1460-2075.1995.tb07185.x PMC398292

[B29] Giulian D Baker TJ Characterization of ameboid microglia isolated from developing mammalian brain J Neurosci 1986 6 2163 2178 3018187 10.1523/JNEUROSCI.06-08-02163.1986 PMC6568755

[B30] Saura J Tusell JM Serratosa J High-yield isolation of murine microglia by mild trypsinization Glia 2003 44 183 189 10.1002/glia.10274 14603460

[B31] Gresa-Arribas N Serratosa J Saura J Sola C Inhibition of CCAAT/enhancer binding protein delta expression by chrysin in microglial cells results in anti-inflammatory and neuroprotective effects J Neurochem 2010 115 526 536 10.1111/j.1471-4159.2010.06952.x 20722966

[B32] Saura J Petegnief V Wu X Liang Y Paul SM Microglial apolipoprotein E and astroglial apolipoprotein J expression in vitro: opposite effects of lipopolysaccharide J Neurochem 2003 85 1455 1467 10.1046/j.1471-4159.2003.01788.x 12787065

[B33] Livak KJ Schmittgen TD Analysis of relative gene expression data using real-time quantitative PCR and the 2(-Delta Delta C(T)) Method Methods 2001 25 402 408 10.1006/meth.2001.1262 11846609

[B34] Buira SP Dentesano G Albasanz JL Moreno J Martin M Ferrer I Barrachina M DNA methylation and Yin Yang-1 repress adenosine A2A receptor levels in human brain J Neurochem 2010 115 283 295 10.1111/j.1471-4159.2010.06928.x 20666933

[B35] Liu YW Tseng HP Chen LC Chen BK Chang WC Functional cooperation of simian virus 40 promoter factor 1 and CCAAT/enhancer-binding protein beta and delta in lipopolysaccharide-induced gene activation of IL-10 in mouse macrophages J Immunol 2003 171 821 828 12847250 10.4049/jimmunol.171.2.821

[B36] Glass CK Saijo K Winner B Marchetto MC Gage FH Mechanisms underlying inflammation in neurodegeneration Cell 2010 140 918 934 10.1016/j.cell.2010.02.016 20303880 PMC2873093

[B37] Sola C Casal C Tusell JM Serratosa J Astrocytes enhance lipopolysaccharide-induced nitric oxide production by microglial cells Eur J Neurosci 2002 16 1275 1283 10.1046/j.1460-9568.2002.02199.x 12405988

[B38] Saura J Angulo E Ejarque A Casado V Tusell JM Moratalla R Chen JF Schwarzschild MA Lluis C Franco R Serratosa J Adenosine A2A receptor stimulation potentiates nitric oxide release by activated microglia J Neurochem 2005 95 919 929 10.1111/j.1471-4159.2005.03395.x 16092928

[B39] Saura J Microglial cells in astroglial cultures: a cautionary note J Neuroinflammation 2007 4 26 10.1186/1742-2094-4-26 17937799 PMC2140055

[B40] Sterneck E Tessarollo L Johnson PF An essential role for C/EBPbeta in female reproduction Genes Dev 1997 11 2153 2162 10.1101/gad.11.17.2153 9303532 PMC275394

[B41] Schroder K Sweet MJ Hume DA Signal integration between IFNgamma and TLR signalling pathways in macrophages Immunobiology 2006 211 511 524 10.1016/j.imbio.2006.05.007 16920490

[B42] Meir O Dvash E Werman A Rubinstein M C/EBP-beta regulates endoplasmic reticulum stress-triggered cell death in mouse and human models PLoS One 2010 5 e9516 10.1371/journal.pone.0009516 20209087 PMC2831074

[B43] Nerlov C C/EBPs: recipients of extracellular signals through proteome modulation Curr Opin Cell Biol 2008 20 180 185 10.1016/j.ceb.2008.02.002 18358708

[B44] Xia C Cheshire JK Patel H Woo P Cross-talk between transcription factors NF-kappa B and C/EBP in the transcriptional regulation of genes Int J Biochem Cell Biol 1997 29 1525 1539 10.1016/S1357-2725(97)00083-6 9570146

[B45] Ejarque-Ortiz A Gresa-Arribas N Straccia M Mancera P Sola C Tusell JM Serratosa J Saura J CCAAT/enhancer binding protein delta in microglial activation J Neurosci Res 2010 88 1113 1123 19908286 10.1002/jnr.22272

[B46] Ramberg V Tracy LM Samuelsson M Nilsson LN Iverfeldt K The CCAAT/enhancer binding protein (C/EBP) delta is differently regulated by fibrillar and oligomeric forms of the Alzheimer amyloid-beta peptide J Neuroinflammation 2011 8 34 10.1186/1742-2094-8-34 21492414 PMC3096570

[B47] Gorgoni B Maritano D Marthyn P Righi M Poli V C/EBP beta gene inactivation causes both impaired and enhanced gene expression and inverse regulation of IL-12 p40 and p35 mRNAs in macrophages J Immunol 2002 168 4055 4062 11937564 10.4049/jimmunol.168.8.4055

[B48] Merrill JE Ignarro LJ Sherman MP Melinek J Lane TE Microglial cell cytotoxicity of oligodendrocytes is mediated through nitric oxide J Immunol 1993 151 2132 2141 8102159

[B49] Chen J Ivashkiv LB IFN-gamma abrogates endotoxin tolerance by facilitating Toll-like receptor-induced chromatin remodeling Proc Natl Acad Sci USA 2010 107 19438 19443 10.1073/pnas.1007816107 20974955 PMC2984206

[B50] Schwartz C Beck K Mink S Schmolke M Budde B Wenning D Klempnauer KH Recruitment of p300 by C/EBPbeta triggers phosphorylation of p300 and modulates coactivator activity EMBO J 2003 22 882 892 10.1093/emboj/cdg076 12574124 PMC145436

[B51] Li H Gade P Nallar SC Raha A Roy SK Karra S Reddy JK Reddy SP Kalvakolanu DV The Med1 subunit of transcriptional mediator plays a central role in regulating CCAAT/enhancer-binding protein-beta-driven transcription in response to interferon-gamma J Biol Chem 2008 283 13077 13086 10.1074/jbc.M800604200 18339625 PMC2442347

[B52] Hanisch UK Kettenmann H Microglia: active sensor and versatile effector cells in the normal and pathologic brain Nat Neurosci 2007 10 1387 1394 10.1038/nn1997 17965659

[B53] Katsuki H Okawara M Shibata H Kume T Akaike A Nitric oxide-producing microglia mediate thrombin-induced degeneration of dopaminergic neurons in rat midbrain slice culture J Neurochem 2006 97 1232 1242 10.1111/j.1471-4159.2006.03752.x 16638023

[B54] Xie Z Wei M Morgan TE Fabrizio P Han D Finch CE Longo VD Peroxynitrite mediates neurotoxicity of amyloid beta-peptide1-42- and lipopolysaccharide-activated microglia J Neurosci 2002 22 3484 3492 11978825 10.1523/JNEUROSCI.22-09-03484.2002 PMC6758387

[B55] Liberatore GT Jackson-Lewis V Vukosavic S Mandir AS Vila M McAuliffe WG Dawson VL Dawson TM Przedborski S Inducible nitric oxide synthase stimulates dopaminergic neurodegeneration in the MPTP model of Parkinson disease Nat Med 1999 5 1403 1409 10.1038/70978 10581083

[B56] Mount MP Lira A Grimes D Smith PD Faucher S Slack R Anisman H Hayley S Park DS Involvement of interferon-gamma in microglial-mediated loss of dopaminergic neurons J Neurosci 2007 27 3328 3337 10.1523/JNEUROSCI.5321-06.2007 17376993 PMC6672486

[B57] Chen H Jacobs E Schwarzschild MA McCullough ML Calle EE Thun MJ Ascherio A Nonsteroidal antiinflammatory drug use and the risk for Parkinson's disease Ann Neurol 2005 58 963 967 10.1002/ana.20682 16240369

[B58] Gao X Chen H Schwarzschild MA Ascherio A Use of ibuprofen and risk of Parkinson disease Neurology 2011 76 863 869 10.1212/WNL.0b013e31820f2d79 21368281 PMC3059148

[B59] in't Veld BA Ruitenberg A Hofman A Stricker BH Breteler MM Antihypertensive drugs and incidence of dementia: the Rotterdam Study Neurobiol Aging 2001 22 407 412 10.1016/S0197-4580(00)00241-4 11378246

[B60] Sterneck E Johnson PF CCAAT/enhancer binding protein beta is a neuronal transcriptional regulator activated by nerve growth factor receptor signaling J Neurochem 1998 70 2424 2433 9603207 10.1046/j.1471-4159.1998.70062424.x

